# Towards Naples Ecological REsearch for Augmented Observatories (NEREA): The NEREA-Fix Module, a Stand-Alone Platform for Long-Term Deep-Sea Ecosystem Monitoring †

**DOI:** 10.3390/s20102911

**Published:** 2020-05-21

**Authors:** Emanuela Fanelli, Jacopo Aguzzi, Simone Marini, Joaquin del Rio, Marc Nogueras, Simonepietro Canese, Sergio Stefanni, Roberto Danovaro, Fabio Conversano

**Affiliations:** 1 Department of Life and Environmental Science, Polytechnic University of Marche, 60131 Ancona, Italy; r.danovaro@univpm.it; 2 Stazione Zoologica Anton Dohrn, 80121 Naples, Italy; jaguzzi@icm.csic.es (J.A.); simone.marini@sp.ismar.cnr.it (S.M.); simonepietro.canese@szn.it (S.C.); Sergio.stefanni@szn.it (S.S.); fabio.conversano@szn.it (F.C.); 3 Instituto de Ciencias del Mar, CSIC, 08003 Barcelona, Spain; 4 Institute of Marine Sciences, CNR, 19032 La Spezia, Italy; 5 SARTI Research Group, Electronics Department, Universitat Politècnica de Catalunya, 08800 Vilanova i la Gertru, Spain; joaquin.del.rio@upc.edu (J.d.R.); marc.nogueras@upc.edu (M.N.)

**Keywords:** stand-alone observatory, optoacoustic imaging, ecological monitoring, remote data transmission, Artificial Intelligence

## Abstract

Deep-sea ecological monitoring is increasingly recognized as indispensable for the comprehension of the largest biome on Earth, but at the same time it is subjected to growing human impacts for the exploitation of biotic and abiotic resources. Here, we present the Naples Ecological REsearch (NEREA) stand-alone observatory concept (NEREA-fix), an integrated observatory with a modular, adaptive structure, characterized by a multiparametric video-platform to be deployed in the Dohrn canyon (Gulf of Naples, Tyrrhenian Sea) at ca. 650 m depth. The observatory integrates a seabed platform with optoacoustic and oceanographic/geochemical sensors connected to a surface transmission buoy, plus a mooring line (also equipped with depth-staged environmental sensors). This reinforced high-frequency and long-lasting ecological monitoring will integrate the historical data conducted over 40 years for the Long-Term Ecological Research (LTER) at the station “Mare Chiara”, and ongoing vessel-assisted plankton (and future environmental DNA-eDNA) sampling. NEREA aims at expanding the observational capacity in a key area of the Mediterranean Sea, representing a first step towards the establishment of a bentho-pelagic network to enforce an end-to-end transdisciplinary approach for the monitoring of marine ecosystems across a wide range of animal sizes (from bacteria to megafauna).

## 1. Introduction

Marine ecosystems drive global biogeochemical cycles and fuel a large part of the Earth’s climatology and food webs [1]. The huge biological complexity characterizing marine communities makes the development of well-resolved and ecologically robust models particularly challenging. Marine assemblages are composed by species spanning their size over several orders of magnitude within all kingdoms of life. Approaching ecosystem complexity requires an adaptive integration between data acquisition and data-analysis tools, covering established environmental observations (i.e., physical, geo-chemical) with innovative sampling approaches (i.e., optoacoustic and environmental DNA-eDNA based) over different temporal frequencies and durations [2,3,4].

The data collected require complex processing by advanced computational approaches, such as niche and network modelling, as well as Artificial Intelligence (AI) algorithms [5,6], thus extending a vision already introduced in plankton investigations [7,8]. In addition, marine ecosystems are increasingly subjected to different anthropogenic stressors that can act synergistically and alter their diversity and functioning in a still, to date, not fully understood fashion. Identifying and quantifying the effects of those pressing threats requires an integrative approach encompassing all marine sciences and all level of biological organizations from genes to ecosystems [9]. Addressing such pressures requires long-time series of bio-ecological data and an integrative approach for their analysis (e.g., Station M, https://www.mbari.org/station-m-time-series/ as well as Porcupine Abyssal Plain-PAP station, https://projects.noc.ac.uk/pap/).

### 1.1. Ocean Observatories

A scenario of increasing exploitation of marine biotic and abiotic resources (e.g., fisheries, oil and gas extraction, and mining) urgently imposes the development of novel monitoring technologies, beyond the traditional vessel-assisted, time-consuming, high-cost sampling surveys [10].

The implementation of fixed observatories, possibly equipped with docked mobile platforms, for the seabed and water column investigation and monitoring are presently enforced, to cooperatively measure biological features and environmental (physical-chemical) parameters [2,11]. Video and acoustic imaging are becoming central approaches for studying benthic megafauna, quantifying species presence (as proxy of local abundance), compiling richness lists, and filling up ecological knowledge gaps on their behavioral interactions (and hence picturing the trophic architectures) in a remote, continuous, and prolonged fashion (e.g., [12,13,14]).

Under this vision, seafloor observatories (cabled or stand-alone) are a substantial innovation in marine monitoring technology (e.g., [15,16,17]). These observatories allow the remote, real-time, and continuous monitoring of marine ecosystems at virtually all depths [2]. The key to their success in environmental monitoring as observatory multi-sensor platforms is in their connectivity to the shore by a cable providing power and real-time data transmission to and from the observatory. To date, benthic networks of those platforms have been established in different oceans, as the Ocean Observing Initiative (OOI), the Ocean Network Canada (ONC) or the EMSO (European Multidisciplinary Seafloor and Water Column Observatory Network), among others [18]. However, deployment of cabled infrastructures is not always viable because of the high cost and, especially in deep sea, the large distance from the putative deployment site and the coast could increase costs and logistical constrains [19]. Stand-alone platforms could represent in this sense a suitable alternative, capable of transmitting data in quasi-real or even real time according to the transmission method selected.

Infrastructural networks envisaging the deployment of cabled observatories and stand-alone infrastructures are transforming the research of the oceans, by establishing a system of interactive, globally distributed sensors with real-time data acquisition [2]. However, the monitoring of several biodiversity components cannot be detected by optoacoustic imaging or other more classic vessel-assisted sampling methodologies (e.g., trawling, or Remotely Operated Vehicles’ video transecting) as in the case of bacteria, meio- and macrofauna. Moreover, the determination of species presence is a problem related to their spatio-temporal traceability beyond the reach of local monitoring nodes with their data collection procedures (e.g., animals passing by, too distantly or when sensors are off). Therefore, there is the need to augment marine observatories with novel bio-ecological sensing and data processing technologies and know-how.

### 1.2. The Strategic Need for a New Ecological Monitoring of the Deep Sea

In the last years, projects and initiatives like EUROCEANS or GOOS (Global Ocean Observing System) (and its deep sea declination DOOS [20]) have set the basis for the definition of essential ocean variables and development of a network of ocean observatories, allowing the recording of key variables at fixed geographical locations in the ocean, and providing in situ measurements, from the surface to the seafloor every few hours, over long periods. Moreover, in order to include the analysis of stressors (e.g., habitat integrity, pollutants, plastics, etc.), in assessing deep-sea ecosystem health, a set of essential biological and ecological variables have been recently identified and prioritized for deep-ocean monitoring [8]. Some deep-sea areas, that act as hot-spots of biodiversity and/or that play a fundamental role in shelf-slope connectivity, like submarine canyons, could be prioritized in such monitoring, as they can deeply affect the overall ecological status of continental margins (e.g., [21,22]). Through submarine canyons, coastal and deep-sea ecosystems are strictly connected by upwelling, downwelling, currents and other wind-driven processes [23]. Submarine canyons are also active corridors for transport of materials and organisms from the land into deep waters, via oceanographic drivers or via exceptional meteorological events such as cascading [24]; they are hotspots of biodiversity, nursery areas and juvenile shelters [25,26]. Thus, the environmental status and resources of the coastal zones are linked to deep-sea ecosystems through these bi-directional exchanges of materials, nutrients, contaminants, and organisms [22,27,28,29,30]; changes in one system may, therefore, influence others. 

Another important aspect to be considered in deep-sea ecological monitoring is the strong variability in sampling of marine species, communities, and overall biodiversity, as result of animal behavior taking place over different spatial scales of depth and latitude, as well as over various temporal scales and encompassing periodicities such as, for example, the day–night cycle and seasonal variations [31,32,33,34]). Rhythmic behaviors, such as population day–night and seasonal displacements take place in a wide variety of marine organisms including cetaceans, fishes and crustaceans [33,35]. Classic sampling techniques such as trawling may produce biased pictures of population size and structure (i.e., class-size and sex-ratio) if not repeated in one area at an hourly frequency over multiple yearly cycles (e.g., [36,37]). Thus, the monitoring of activity rhythm is of relevance for an advanced understanding of their effects on ecosystem functioning and productivity [4,10].

### 1.3. Objectives

**Figure 1:**
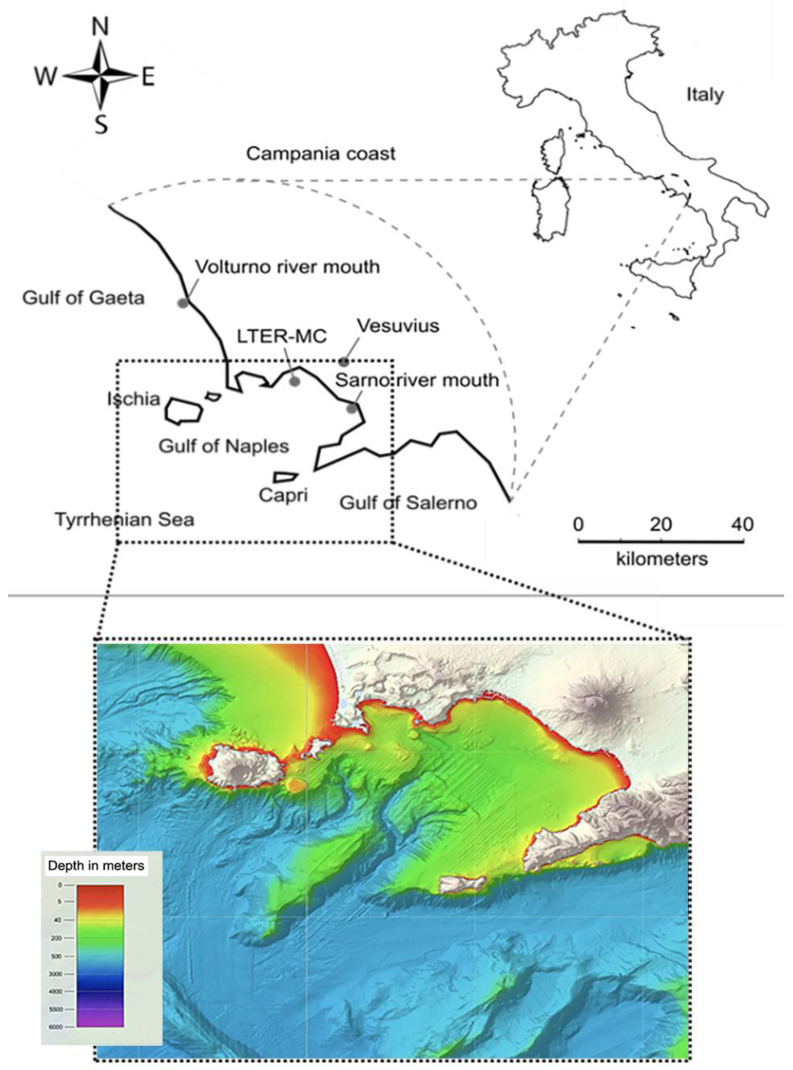
Map of the study area (**top**) with the Gulf of Naples and the LTER-MC station in front of Naples along with the EMODNET bathymetry of the Dohrn Canyon (**bottom**) where NEREA-fix platform will be deployed.

Here, we present the concept and design of NEREA-fix, a new stand-alone observatory that will encompass sensing technologies in the deep-sea ecosystem of the Dohrn Canyon (Gulf of Naples, Tyrrhenian Sea). NEREA-fix will be part of the whole NEREA infrastructure that encompasses also the Long Term Ecological Research (LTER) station of “Mare Chiara” (MC), located ca. 2 nm off Naples coast (Figure 1, top), at 80-m depth, and a series of periodical water and plankton sampling within the whole area (NEREA-mob, see below). NEREA-fix aims at representing an off-shore, long-term monitoring station for the study of deep-sea biodiversity and ecosystem functioning off a demographically dense urban area, subjected to multiple environmental stressors including seismic activity and global climate change. NEREA-fix also aims at providing data to complement the knowledge on the local ecosystems tackling life components with a range of sizes from macro- to megafauna.

## 2. Deployment Characteristics

### 2.1. The Deployment Area

For NEREA-fix deployment we selected the Gulf of Naples (GoN), a roughly 15 km long gulf located along the south-western coast of Italy, bordered by the Naples and Pozzuoli urban areas and Mount Vesuvius. The GoN is closed by Capri, Procida and Ischia islands. The Naples metropolitan area accounts for ca. 4.5 million inhabitants and the entire population is distributed along a very narrow area along the coast. The presence of dumping and untreated sewage, along with the proximity to historically contaminated coastal areas, the intense maritime transportation, the concentration of maritime infrastructures (ports and coastal defenses), and other direct and indirect anthropogenic stressors [38,39,40,41] and the presence of degraded marine habitats [42], make this area of particular interest for the study of multiple stressors in the deep sea.

The water mass structure of the GoN is predominantly linked to the main circulation of the southern and mid-Tyrrhenian Sea, with influences from local factors, such as the wind stress and the river runoff. The two main water masses flowing in the GoN are the Modified Atlantic Water (MAW) that occupies the upper 50–100 m and the Levantine Intermediate Water (LIW) located below ~200–300 m [43,44]. Depending on the season, other water masses can be recognized within the GoN, such as the Tyrrhenian Intermediate Water (TIW), formed during winter mixing at depths down to ~150 m, and the Tyrrhenian Surface Water (TSW), found above 75 m as the result of summer warming and freshening of the TIW [45]. The main submarine feature of the GoN is the Dohrn Canyon, a bifurcate structure (see Figure 1, bottom) that indents perpendicular to the coastline the continental shelf 12 nm off Naples, beginning at ca. 250 m and sharply declining down to ca. 1300 m in the Tyrrhenian plain [46].

Concerning the ocean state of the area, an analysis of the wind intensity and direction data and wave height of the Dohrn Canyon area was carried out by SZN, to estimate the prevailing winds and the maximum wave height during the year. The seasonal average values showed a wind distribution on the four main quadrants, with a prevalence of winds from SE and SW (sirocco and libeccio winds) and a maximum average speed of about 5 m/s (https://www.ecmwf.int/en/forecasts/datasets). Significant wave height of the sea surface shows seasonal trends with average values of 1–7 m (https://resources.marine.copernicus.eu). This information was useful in identifying the best characteristics of the buoy, the anchoring and the cabling system of the seabed platform.

Very little information exists regarding the biological communities of the area; a recent study highlighted the occurrence of a unique and novel biotope formed by deep-water corals and bivalves (*Acesta excavata* and *Neopycnodonte zibrowii*) in the canyon walls [42]. Additionally, demersal nursery areas for the European hake (*Merluccius merluccius*), the blackmouth catskark (*Galeus melastomus*), the deep-water rose shrimp (*Parapenaeus longirostris*), the horned octopus (*Eledone cirrhosa*) have been evidenced within the Gulf [47].

### 2.2. NEREA Research Infrastructure

NEREA is based in the GoN and encompasses three elements: (i) a coastal station, i.e., the LTER-MC; (ii) a stand-alone deep platform in the Dohrn Canyon (NEREA-fix); and (iii) NEREA-mob [48]. The LTER-MC has been sampled for plankton and other parameters since 1984 and its data set represents one of the few plankton time series available in the Mediterranean Sea, and one of the longest [49]. NEREA is led by Stazione Zoologica Anton Dohrn of Naples (SZN), with the collaboration of Italian and Spanish universities and institutes (Polytechnic University of Marche and Institute of Marine Sciences-CNR from Italy and Polytechnic University of Catalunya and Institute of Marine Sciences-CSIC from Spain).

**Figure 2:**
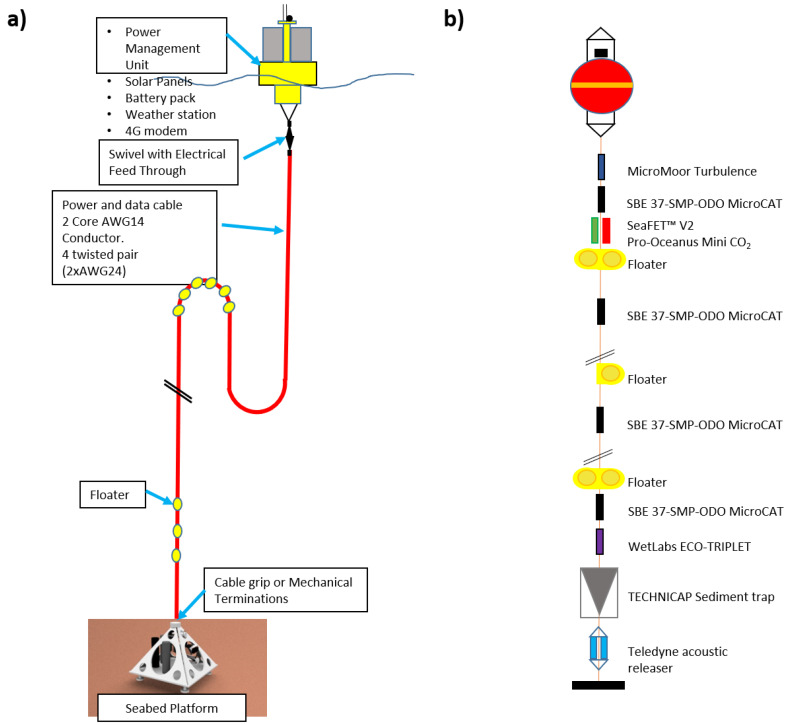
Configuration scheme and sensors of (**a**) the Seabed Platform (SP) and (**b**) the Mooring Line (ML).

**Figure 3:**
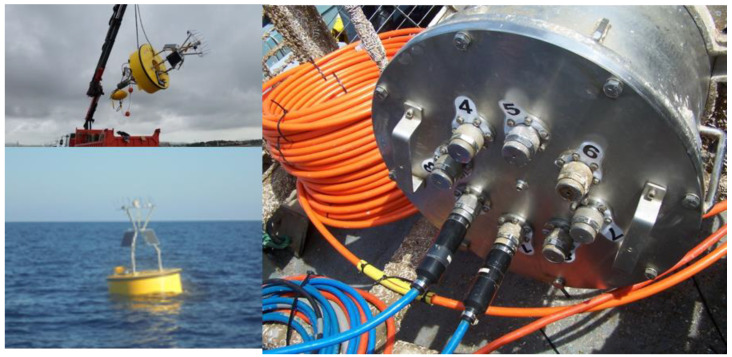
The used moored surface buoy (**on the left**) and depth-rated SP junction box (**on the right**) for motherboard processing and sensors connections.

Here, the NEREA-fix, consisting of a mooring line (ML) in stand-alone mode, positioned close to a seabed platform (SP) installed on the bottom at a depth of 650 m (Figure 2a,b) and connected, instead, to a surface buoy (Figure 3), will be deployed by autumn 2020.

### 2.3. The Seabed Platform (SP), the Mooring Line (ML) and the Surface Buoy

The Seabed Platform (SP) will allocate the junction box (OBSEA-like; www.obsea.es; [14]), containing the motherboard for sensors management, data acquisition and embedded routines for image preprocessing (see next Section).

The SP will be connected to the surface buoy through a power data cable, providing energy for cameras, sensors and the motherboard (Figure 2a). An Ethernet fast connection for data transfer and communication between the surface and the SP will be established adopting a couple of Ethernet-over-twist pair extenders in redundant configuration, thus allowing to reach a bandwidth of 100 mbps.

A kevlar cable grip or a mechanical termination will secure the sea cable to the SP and the surface buoy; furthermore, a swivel with an electrical feed through installed below the buoy will eliminate stress due to buoy rotations. The surface buoy will transmit real-time data to the shore station using the mobile network 4G (upgradable in the future to 5G). Communication tests have been carried out in the area to ensure connectivity, but in the event of a temporary lack of coverage, data can be stored in a buffer for later transmission. All the power required by the SP will be generated by the solar panels and stored in the batteries of the surface buoy, thus with the power coming directly from the surface there will be no need of batteries in the seabed. Therefore, the whole structure will be recovered only for cleaning, sensor calibration or substitute components, and maintenance operations could be planned every 12–24 months. In order to achieve this maintenance period, all the optical sensors will be protected with active anti-fouling systems (UV light, wipers or local chlorination systems); in addition, a post-campaign sensor calibration will provide sensor drift information for data postprocessing. Furthermore, the SP junction box will have enough storage capacity to archive the entire dataset allowing in this way to retrieve the information in case it cannot be transmitted to the shore station For the maintenance operation, an acoustic release will free the weight, letting the structure rise to the surface.

The proposed buoy will be a toroidal shape, 2 m in diameter (see Figure 3) that will host solar panels for the dry sensors, a meteorological station (for the acquisition of data on wind direction and speed, air temperature, atmospheric pressure), GPS receiver and tracker, and communications electronics.

### 2.4. Instruments and Sensors

Technical details of the oceanographic, geochemical and bio-ecological sensors to be mounted on the SP and ML are provided in Table 1 and also detailed in Figure 2.

Concerning the sediment trap on the ML, this will allow the measurement of the mass flux and the Organic Carbon flux (together with the turbidimeter), along with the Benthic Boundary Layer (BBL) zooplankton and its trophic role in the benthopelagic coupling according to [50,51]. The Acoustic Doppler Current Profiler (ADCP) on the ML will serve for both current measures and diel vertical migration estimation, according to [52]. Acoustic releases will allow the periodical (i.e., annual) recovery of the mooring for maintenance operations and successive re-deployment.

The environmental multiparametric data, acquired at both the SP and the ML, will be coupled with image data collection (on the SP) to assess species richness and local variations in counts as a proxy for the status of their abundance, and how such a variation is related to oceanographic and geochemical flux constrains [2,11]. A color camera will acquire images using a 5600 k and 2750 lm LED light (see Table 1). Light has been shown to disrupt deep-sea animal visual systems [53] and artificial photic stimulation may attract animals to benthic infrastructures [30]. Light at filming should, therefore, be avoided, and optoacoustic technologies may be used to reduce the monitoring footprint, by using soundwaves instead of photons to compose images (2,4). The imaging radius will be expanded beyond the visible range with a concomitant multibeam-based acoustic imaging (following protocols specified by [2,4] by an Adaptive Resolution Imaging Sonar (ARIS) camera (see Table 1) [54]. The ARIS can visualize fish and invertebrate shapes in the dark and track the movement of individuals at distances greater than those achieved by visual systems equipped with artificial lighting solutions, recording their projection in a water column, tens of meters away depending on the angle of the form-emitting sound cone [54]. A limitation of acoustic camera use, however, is related to animal identification, which is solely based on morphology (i.e., no colorimetric and limited texture information is captured). Accordingly, in order to verify acoustic identifications, multibeam cameras must be deployed simultaneously with optical imaging equipment for preliminary richness cross-validation [2]. The time-lapse for the video and acoustic cameras will set up according to the ecology of the targeted deep-sea species [4]. Setting the correct sampling design is fundamental to mirror the actual species diversity and abundance pattern, although at a low spatial scale, as that provided by fixed-point observation.

Thanks to the two-way communication system between Shore Station and Seabed Platform it will be possible to modify sampling frequency and some configuration parameters to adapt the system to real present conditions.

All the sensors incorporated within the platform will operate at high frequency (e.g., making measurements each 30 min; see [30,55] for a procedural example) with multiparametric environmental data acquisition carried out over consecutive years, covering seasonal reproductive periods, periodic movements and migrations, and molting processes [56]. The NEREA SP will be also equipped with an embedded processing unit (e.g., Jetson TX2 Module) for performing simple data analysis tasks interpreting the content of the acquired data [5,57].

The sensing technology in the NEREA observatory will be compliant with the Sensor Web Enablement standards [58] and, based on this, intelligent services will be studied and developed for an adaptive management of the data collection [5,59]. NEREA-fix provides an almost real-time data transmission system. In case of the absence of the 4G signal, the data packet will be temporarily stored on a memory unit of the control/transmission system installed on the surface buoy (512 GB capacity). As soon as the signal is available, the system will automatically transmit the data to a dedicated server; this will be possible through a graphical interface to check the levels of the batteries which power the infrastructure and that the instruments are functioning correctly.

### 2.5. The Coupled Data Acquisition by NEREA-Fix and NEREA-Mob and Omics Expansion

The NEREA-fix will adopt an end-to-end transdisciplinary approach for the study of marine ecosystems, towards the establishment of augmented observatories concept (i.e., by adding omics data to in situ sampled organisms). Data science methodologies for modelling, understanding, monitoring and forecasting environmentally relevant phenomena will be developed based on the integrated data collected [60]. For this reason, NEREA-fix monitoring activity will be coordinated with sampling procedures from the already existing NEREA-mob, which encompasses periodical sampling at sea with oceanographic vessel from the LTER Mare Chiara to the off-shore area including the water column above the Dohrn Canyon (see Figure 1).

A flexible platform cyberinfrastructure has been conceived to host specific modules for data management and bioinformatics (NEREA-dat), and modelling (NEREA-mod), the latter being a module in which ecological and physical data will be integrated into mesoscale circulation models. NEREA-fix infrastructure will be further enhanced through the creation of an exchange information system that will allow data sharing, with access by a large community of users and also by an outreach and dissemination system in order to reach a wider audience in the context of Citizen Science protocols. We will focus on the data management quality (data and metadata availability and access to users, standard/regular QC/QA procedures, according to FAIR principles) and a qualified team has been already formed for the observatory operation, maintenance, development, management according to the standards required by EMSO-ERIC.

In particular, data analysis algorithms, obtained through supervised and unsupervised machine learning approaches will be developed and executed on the processing unit embedded on the SP. These algorithms will be used for recognizing and classifying the macrofauna- and megafauna-relevant subjects contained in the acquired optical and acoustic images. Images without any relevant content will be discarded [61,62] without being communicated at the land station in order to reduce data transfer loads. Content-based image analysis algorithms will be learned at the land laboratory based on images acquired by the SP. The learning process will be based on ground truth datasets labeled by experts, and the resulting algorithms will be transferred to the SP through the double-direction communication facility. On land, automated procedures for the multivariate analyses and modeling of time-series combining environmental, biological, biogeochemical, and metabarcoding data will be used for understanding ecosystem dynamics [63,64].

## 3. Discussion

Cabled seafloor observatories are among the most substantial recent innovations in marine technology since they allow the remote, real-time, continuous and long-term monitoring of marine ecosystems at virtually all depths [2]. Acoustic and imaging technologies, combined with data processing, can provide remarkable insights into the state of marine ecosystems and the level of experienced impacts [4]. Unfortunately, the key to their success in environmental monitoring is their connectivity to the shore by a cable providing power and real-time data transmission. Cabled observatories cannot be moved and, therefore, are not sufficient for defining the conditions of ecosystem management. At the same time, the deployment of these high-cost cabled infrastructures is not viable in some locations of interest as canyon axes or in areas where commercial trawl often occurs (i.e., due to the possible damage caused by fishing activities to the cable). Specifically, the design, construction, and deployment of a cabled observatory far away from shore could be one order of magnitude higher than that of a stand-alone platform, due to the cable cost and its laying costs (from 500 k€ to 5 M€).

For all these characteristics, the connection of those platforms with the Marine Strategy Framework Directive (MSFD, 2008; [65]) is still under development, since the spatial component of analysis is also crucial. The MSFD Directive was developed to achieve the good environmental status in marine ecosystems. Given the complex spatio-temporal dimension of anthropogenic impacts, the development of reliable and environmentally global monitoring strategies for the marine ecosystems is urgently required.

In this endeavor, the technological development of environmental sampling strategies which ensure that data are collected with a frequency, continuity and complexity to best address the management questions is fundamental. Thus, multiparametric, intelligent (i.e., smart) platforms represent the future for European marine technological innovation; imaging and acoustic multiparametric technologies could be successfully used as powerful instruments to characterize the marine environment, its biomass, biodiversity, detecting and providing estimates of pollution and marine litter, being therefore of importance to supporting marine environments and monitoring fisheries [2]. Such monitoring efficiency is closely linked to the duration and frequency of data acquisition, with the technological development towards the construction of stand-alone observatories like the one presented here, capable of autonomous broad oceanographic and biological monitoring at a high frequency (i.e., minutes) over long temporal durations (i.e., months), and with easily movable capabilities (i.e., spatially non constrained). At the same time, data spatial interpolation for the scaling could be developed to increase ecological representation of local monitoring results (e.g., [66,67,68]). Finally, in the future, additional sensors, such as PAM for cetaceans or underwater noise monitoring should be added, as the area is of crucial importance for marine mammals [69] and also characterized by intense maritime traffic.

## 4. Towards a Network of Deep-Sea Augmented Observatories

Disentangling the effect of natural variations from those generated by anthropogenic changes is crucial to quantify and then predict the magnitude and impact of future climate changes. Human activities and global change are exacerbating coastal eutrophication, expanding hypoxic and anoxic areas and modifying pH, biogeochemical cycles, with potentially dramatic consequences on marine life and ecosystems. Deep-sea ecosystems are not spared by global impacts and species loss, and habitat destruction is severely altering an increasing portion of deep-sea ecosystems [9,10]. Such a scenario requires a close monitoring of the changing properties of the oceans and the effects of potential shifts on marine species and habitats. This requires a continuous stream of high-quality data on climatically and ecologically relevant variables at different key locations. In this context, multidisciplinary underwater observatories provide real, high-temporal resolution data in real or quasi-real time, depending on the type of transmission, and allows to better understand short-term variations and ecosystem dynamics. This fact highlights the importance of deep-sea observatories in the current international effort to ocean monitoring. These observatories can acquire large amounts of high-resolution data and are able to analyze annual tendencies and singular events, providing new opportunities of research and development in research projects as well as technological innovation. The integration of omics technologies represents the next step towards incremental measurement capabilities by robotic platforms, which would acquire the capability to sense the entire marine community within a wide range of ecological sizes within a single sample and beyond the reach of optoacoustic field of views.

Indeed, a way to validate the biodiversity recorded on video imaging, as well as the integration of those organisms not spotted on cameras, is the integration of the complex mixture of environmental genomic DNA (eDNA) and RNA (eRNA) belonging to many different organisms found in an environmental sample [70]. In recent years, a growing need to achieve a global characterization of biodiversity in marine ecosystems has motivated the development of omics technologies [2]. Considering the undergoing loss of biodiversity, we need to take up the challenge to develop alternative, less impactful monitoring methods. Beyond research in ecology, eDNA/eRNA has also been proven to be a useful material for biodiversity monitoring purposes [71]. DNA-based taxonomic or functional information is possible for the ecosystem under consideration. Its use is revolutionizing the way we assess biodiversity and has the potential to change practices and policies in management and conservation [72,73,74,75]. Combining eDNA/eRNA with metabarcoding protocols—better when targeting multiple markers—has proven to be very effective in assessing marine diversity at a high resolution [76]. Several studies have demonstrated that eDNA metabarcoding recovers higher diversity of taxa than other sampling methods (i.e., [77,78]). Moreover, a few cases have proven eDNA suitable for quantitative assessment (as relative species abundance) of fishes [79,80]. Therefore, the combined approach of a vision-based ecosystem assessment integrated with an eDNA metabarcoding should provide a more holistic view of marine communities.

The creation of an eco-genomic sensor device would dramatically increase the biological spatio-temporal monitoring capability of cabled observatories and mobile platforms [2], including vessels [81], favoring the transition of EMSO infrastructures toward their augmented status, serving at the same time as inspiration for other relevant networks as ONC and OOI.

Moreover, given the potential cumulative impacts of multiple stressors (i.e., trawling, mining, etc.) on deep-sea ecosystems, it is also urgent to identify deep-sea conservation priorities and, specifically, in the context of deep-sea observatories, which are the key-areas where these infrastructures need to be placed first, thus introducing a spatial perspective. Recent analyses of spatial planning extended to the deep-Mediterranean Sea [82] are supporting the definition of guidelines for deep-sea ecosystem and biodiversity conservation [83] in the Mediterranean basin, and the choice of NEREA and other new observatories will allow the principles outlined in these works to be incorporated.

## 5. Conclusions

Considering the growing human pressure on marine ecosystems, the assessment of the ‘Ocean Health’ (OH) should be planned as follows: (i) well routed on scientific tools, quantitatively characterizing target key elements of the ocean’s status, including biological, physical, economic and social ones, and (ii) devoted to guide decision makers towards the sustainable use of marine resources. Non-cabled and intelligent multiparametric observatories technology has yet to be implemented. Such technology has relevance for the Blue Growth call, which requires the development of acoustic and imaging technologies to strengthen marine industry, support the global monitoring of the marine environment (seabed and water column characterization), as well as fishery policies and ocean discovery. In the future, a network of augmented observatories coupling plankton and benthos, using an end-to-end approach (from microbes to large marine mammals) will enable to effectively assess OH. Such a network will operate under a multidisciplinary context, encompassing the simultaneous measurement of biogeochemical and oceanographic variables and will be managed by Sensor Web Enablement and Internet of Things services and data science approaches. These data will support knowledge-based management toward the achievement of Sustainable Development Goal 14. In this framework, the NEREA initiative is a new technology-based, analytical platform for such an OH-oriented assessment, integrating existing cutting-edge technologies and implementing near future ones (i.e., omics and eDNA), allowing a broad ecological monitoring of marine ecosystems, and with applications within the maritime industry; NEREA will also, in the near future, shed new light in the marine science panorama and produce new knowledge for improving ecosystem services.

## Figures and Tables

**Table 1:** Mooring line (ML) and Seabed platform (SP) oceanographic instruments integration.

**Mooring Line (ML)**		
**Num. of Units**	**Sensor/Probe/Tool**	**Description**
4	SBE 37-SMP-ODO MicroCAT (positioned at 4 different depths along the vertical)	Integrated measurements of depth, temperature, salinity and dissolved oxygen
1	MicroMoor Turbulence Measurement Mooring	Measurement of turbulent flow in the marine environment
1	Pro-Oceanus Mini CO_2_	CO_2_ estimate
1	SeaFET™ V2	pH estimate
1	WetLabs ECO-TRIPLET	Integrated measurements of fluorescence, CDOM (Colored Dissolved Organic Matter) and turbidity (above the sediment trap)
1	TECHNICAP Sediment trap	Determination of downward mass fluxes of particles and associate elements
2	Teledyne acoustic releaser	Recovery of mooring
**Seabed Platform (SP)**		
**Num. of Units**	**Sensor/Probe/Tool**	**Description**
1	Deep SeapHOx™ V2	Integrated measurements of depth, temperature, salinity, pH and dissolved oxygen
1	WHLS75-Long Ranger 75 kHz Self-Contained ADCP (Sentinel Configuration)	For gathering detailed data on seasonal and annual current structure fluctuations
1	WetLabs ECO-TRIPLET	Integrated measurements of fluorescence, CDOM and turbidity
1	Pro-Oceanus Mini CO_2_	CO_2_ estimate
1	ARIS Voyager 3000 System	Dual-frequency identification sonar (DIDSON) technology with the release of high-resolution and high definition imaging sonars
1	LUXUS Colour Zoom Camera II and LUXUS Power LED	720p video camera
1	TRIOS RAMSES PAR sensor	Hyperspectral radiometer
